# Reward-Related Attentional Capture Is Associated With Severity of Addictive and Obsessive–Compulsive Behaviors

**DOI:** 10.1037/adb0000484

**Published:** 2019-06-20

**Authors:** Lucy Albertella, Mike E. Le Pelley, Samuel R. Chamberlain, Fred Westbrook, Leonardo F. Fontenelle, Rebecca Segrave, Rico Lee, Daniel Pearson, Murat Yücel

**Affiliations:** 1 Turner Institute for Brain and Mental Health, Monash University; 2 School of Psychology, UNSW Sydney; 3 Department of Psychiatry, University of Cambridge; 4 School of Psychology, UNSW Sydney; 5 Turner Institute for Brain and Mental Health, Monash University, and Obsessive, Compulsive and Anxiety Research Program, Institute of Psychiatry, Federal University of Rio de Jeneiro; 6 Turner Institute for Brain and Mental Health, Monash University; 7 School of Psychology, UNSW Sydney; 8 Turner Institute for Brain and Mental Health, Monash University

**Keywords:** reward learning, compulsivity, addiction

## Abstract

A cue that signals reward can capture attention and elicit approach behaviors in people and animals. The current study examined whether attentional capture by reward-related cues is associated with severity of addiction-related and obsessive–compulsive behaviors. Participants were recruited via Mechanical Turk and included 143 adults (*M*_age_ = 34 years, *SD* = 8.5; 43% female) who had endorsed at least 1 addiction-related or obsessive–compulsive behavior in the past month. All assessment components were delivered via the Internet and included questionnaires to assess severity of compulsivity-related problems across addiction-related and obsessive–compulsive behaviors, as well as a visual search task to measure reward-related attentional capture. Reward-related attentional capture was associated with severity of compulsivity, transdiagnostically. These findings have implications for understanding the mechanisms that underlie compulsive behaviors and suggest that reward-related attentional capture is a promising transdiagnostic cognitive risk marker for compulsivity.

Individuals who use psychoactive substances excessively, or who have been diagnosed with a substance use disorder, typically show an attentional bias toward stimuli associated with that substance (Cousijn et al., 2013; Field & Cox, 2008; Lubman, Peters, Mogg, Bradley, & Deakin, 2000; Nikolaou, Field, & Duka, 2013). Such biases have been long-thought to form as a result of the associations produced by drug use. Specifically, through repeated pairing of drug-related stimuli and the rewarding effects of taking the drug, those previously neutral stimuli acquire incentive salience, subsequently attracting attention and evoking approach responses in their own right (Berridge, Robinson, & Aldridge, 2009; Robinson & Berridge, 2000).

However, a growing body of research suggests that there is variability in the likelihood that individuals attribute incentive salience to signals of reward, and hence in the ability of such signals to capture attention and evoke approach. The ability of reward-predictive cues to direct behaviors toward themselves has been well-documented in Pavlovian conditioning studies in animal subjects, a phenomenon termed *sign tracking* (Boakes, 1977; Hearst & Jenkins, 1974). Moreover, the study of this phenomenon has revealed individual differences that are related to drug addiction. Specifically, some rats approach and contact a lever that signals the arrival of food (sign trackers), whereas other rats learn to approach the food magazine (goal trackers) when the lever is presented. The sign trackers treat the lever as if it were the food (the lever has acquired incentive salience as well as signal value), whereas the goal trackers use the lever to tell them when to approach the magazine (the lever has acquired signal value only). Critically, the sign trackers are more likely to become addicted to drugs, such as cocaine, than the goal trackers (Flagel, Akil, & Robinson, 2009; Robinson & Flagel, 2009).

These findings suggest that the ability of drug-predictive cues to acquire incentive salience reflects an interaction between drug taking and a preexisting disposition to attribute incentive value to stimuli associated with reward. For instance, that disposition could increase the likelihood that someone will approach and stay longer in contexts related to say alcohol (e.g., pubs), through such locations becoming attractive in their own right rather than as signaling where alcohol can be procured and consumed. Similarly, people with such a disposition (sign-trackers) might find themselves approaching the fridge without intention to eat or attending to food cues even when they are not hungry, in turn being prompted to eat more frequently.

Reward-associated stimuli are not the only stimuli capable of eliciting a sign-tracking response. Safety signals, that is, stimuli that signal the omission of an expected aversive event (such as shock), can also elicit a sign-tracking response (Leclerc & Reberg, 1980). To the extent that sign-tracking reflects the attribution of incentive salience toward Pavlovian cues, the finding that safety signals can elicit a sign-tracking response suggests that, like reward cues, they are also endowed with incentive salience (through signaling the absence of an otherwise expected threat). And, just as there may be individual differences in sign-tracking tendency that reflect a predisposition toward the attribution of incentive salience to cues that signal attractive events, there may also be such differences in attributions of incentive salience to cues that signal the absence of aversive events. It is important to note that sign-tracking toward a safety signal does not imply better or worse safety-signal learning; sign-trackers and goal-trackers may learn about the association between the safety signal and reduced threat equally well. However, although goal-trackers will be likely to use the safety signal to engage in nonthreat-related activities, sign-trackers will instead direct their behaviors toward the safety signal, at the expense of engaging in these other activities. This would mean that individuals predisposed to attribute incentive salience to safety signals could also be at risk of developing cue-triggered, maladaptive behaviors. One instance where such factors may play a role in maintaining symptoms is obsessive–compulsive disorder (OCD). In this disorder, behaviors, such as approaching a sink, turning on taps and washing hands, may initially have served to alleviate anxiety about contamination, thereby becoming safety signals. However, over time, the attribution of incentive salience to the washing-related, safety signals will enable them to command attention, eliciting approach and contact in their own right, even in the absence of any subjective experience of anxiety.

Combining the above ideas, a shared risk factor across addiction-related and obsessive–compulsive behaviors may be the tendency to attribute incentive salience to Pavlovian cues (reward and safety signals, respectively). In fact, enhanced incentive salience attribution processes may underlie compulsive behaviors generally, with compulsive behaviors defined here as repetitive behaviors that are maladaptive in that they cause distress, are difficult to control, and/or are counterproductive to ongoing goals (e.g., Figee et al., 2016; Robbins, Gillan, Smith, de Wit, & Ersche, 2012; Voon et al., 2015).

As noted earlier, individual differences in incentive salience attribution can be assessed in terms of differences in propensity toward sign-tracking. Although research on sign-tracking originated in animal studies, Le Pelley, Pearson, Griffiths, and Beesley (2015) developed a procedure to assess an analogue of sign-tracking in people, specifically via the capture of visual attention. In this task (see Figure 1), participants searched for a diamond target among circles on every trial. The faster they found and responded to this target, the more points they earned (with points converted to money at the end of the experiment). Critically, one of the (nontarget) circles could be colored, either blue or orange (all other shapes were gray). The color of this color-singleton circle—referred to as the *distractor*—predicted the size of the reward available on the current trial: one color (the high-reward color) signaled that a large reward was available, and the other (low-reward) color signaled that a smaller reward was available. Notably, although the distractor signaled reward magnitude, it was not the target that participants responded to receive that reward; thus distractors had a Pavlovian, but not an instrumental, relationship with reward. The key finding was that responses to the target were significantly slower for trials with a high-reward distractor compared to trials with a low-reward distractor. This shows that the high-reward signal was more likely to capture participants’ attention (and hence slow their search for the target) than the low-reward signal. This pattern of greater distraction by the high-reward signal than the low-reward signal—termed *value-modulated attentional capture* (VMAC)—was counterproductive, because it meant participants earned less on high-reward trials than would otherwise have been the case. Thus, just as sign-tracking animals may approach and contact signals for reward even when such approach is at the expense of obtaining the reward, people likewise attend to reward-related cues in the VMAC protocol even when such attending is at the expense of procuring the reward.[Fig fig1]

Sign-tracking in animals has been invoked as a highly promising model of addiction propensity. However, no study to date has explored the potential of attentional capture in the VMAC protocol to serve as a risk marker across compulsive behaviors in humans. The present study did so by evaluating whether greater attentional capture by reward cues is associated with severity of addiction-related and obsessive–compulsive behaviors. We predicted that across behavioral domains (addiction and obsessive–compulsive), participants endorsing greater compulsivity-related problems would show greater reward-related attentional capture. Such a demonstration would support the idea that attentional capture can serve as a transdiagnostic risk marker, and a promising tool to better understand the factors that drive risk for compulsive behaviors across diagnostic boundaries.

## Method

### Ethical Approval and Participants

Adult participants, aged 18 years and above, were recruited via Mechanical Turk for a study advertised as exploring habits and compulsivity, in return for payment of 6 USD.^1^ Each individual provided written informed consent prior to taking part. Participants then completed a series of questionnaires, followed by the VMAC task. Stimulus presentation in all tasks was controlled by Inquisit. All study procedures were carried out in accordance with the human research ethics committee at Monash University, Australia.

Two hundred sixty participants consented to the study. Forty-four participants dropped out before reaching the VMAC task. Thirty-nine participants achieved less than 50% accuracy in this task (i.e., numerically below chance), including participants who did not complete the task in its entirety. Of the remaining sample (*n* = 177), 17 participants had not engaged in an addiction-related or OCD-related behavior in the past month and so did not complete the compulsivity-related problems questionnaire of primary interest in the current study. A further 17, although having consumed alcohol in the last month, reported drinking on fewer than 2 days a week (and reported no other compulsive behaviors). These participants were therefore excluded (though we note that the results remain highly comparable—in direction and significance—if these participants are retained [*n* = 160]). Thus data from 143 participants were included in subsequent analyses.

### Online Survey Measures

Demographic information such as age and gender was collected, and participants completed the following questionnaires:

#### Short UPPS-P Impulsivity Scale

The Short UPPS-P Impulsivity Scale (Cyders, Littlefield, Coffey, & Karyadi, 2014) is a 20-item scale that measures impulsivity with five subscales: Negative Urgency, the tendency toward impulsive action when experiencing strong negative emotions; Positive Urgency, the tendency toward impulsive action when experiencing strong positive emotions; Lack of Perseverance; Lack of Premeditation; and Sensation Seeking. The current study used the total score, a measure of trait impulsivity.

#### Psychological distress

Participants completed the brief Depression Anxiety Stress Scales (DASS-21 Lovibond & Lovibond, 1995). The DASS-21 contains 21 items assessing depression, anxiety, and stress/tension symptoms. The measure of interest was again the total score, reflecting general psychological distress.

#### Brief Assessment Tool for Compulsivity Associated Problems

We developed an assessment to quantify relevant features of a range of compulsive behaviors. Importantly, this assessment aimed to capture experiences and behaviors that applied to a similar extent across addiction and OCD. This feature distinguishes the Brief Assessment Tool for Compulsivity Associated Problems (BATCAP) from other addiction and OCD assessments that have more limited transdiagnostic utility. For example, the Alcohol Use Disorders Identification Test (AUDIT; Saunders, Aasland, Babor, de la Fuente, & Grant, 1993) includes items about driving while intoxicated and binge drinking, which cannot be applied to OCD symptoms; likewise the Yale-Brown Obsessive Compulsive Scale (YBOCS; Goodman et al., 1989) includes items about obsessions, which albeit related, are not central to compulsivity per se. In contrast, BATCAP is transdiagnostic, covering alcohol use, gambling, binge eating, excessive Internet use, as well as contamination, checking, and ordering compulsions. Individuals who reported having engaged in any of these behaviors in the past month were asked to complete the corresponding BATCAP. For each of these behaviors, individuals answered six questions^2^ about time lost, distress, loss of control, functional impact, anxiety if prevented from doing the behavior, and strongest urge (see the online supplementary materials). Items 1 to 5 were adapted from the YBOCS and/or Florida Obsessive-Compulsive Inventory (Storch et al., 2007). Item six was adapted from the Penn Alcohol Craving Scale (Flannery, Volpicelli, & Pettinati, 1999). Each item was rated on a 5-point scale, ranging from 0 (*none/not at all*) to 4 (*extreme/constant*) with the average score for each individual scale calculated. These were then used to calculate two scores: (a) primary addiction score (score of the top-scoring domain across addiction behaviors) and (b) primary OCD score (score of the top-scoring domain across OCD-related behaviors).

We also used the following measures to support the validity of the individual BATCAP scale: AUDIT; Obsessive Compulsive Inventory—Revised (checking, ordering, and washing subscales); Young’s Internet Addiction Test; Problem Gambling Severity Index, and the Binge Eating Disorder Screener. Tables showing correlations are presented in the online supplementary materials.

#### AUDIT

The AUDIT (Saunders et al., 1993) is a 10-item self-report measure developed by the World Health Organisation to assess hazardous/risky alcohol consumption.

#### OIC–R

The OCI–R (Foa et al., 2002) is an 18-item scale assessing six areas of obsessive–compulsive experiences over the preceding month, specifically washing, ordering, checking, obsessing, neutralizing, and hoarding. The present study examined the first three, as these were those assessed by the BATCAP scales.

#### Young’s Internet Addiction Test Short Version

Young’s Internet Addiction Test Short Version (Pawlikowski, Altstotter-Gleich, & Brand, 2013), a 12-item questionnaire, was developed to measure problematic usage of the internet.

#### Problem Gambling Severity Index

The Problem Gambling Severity Index is a nine-item measure of problem gambling severity (derived from the 31-item Canadian Problem Gambling Index (Ferris & Wynne, 2001).

### VMAC—Reward-Only Variant

The visual search task used a reward-only variant of Le Pelley et al.’s (2015, Experiment 2) VMAC procedure, modified to reflect reward-related attentional capture more specifically. In Le Pelley et al.’s original version of the task, participants were punished (by loss of points) for incorrect responses. By contrast, in the current procedure errors did not result in losses. This “reward-only” modification was made to ensure performance was less likely to be confounded by loss-related sensitivity and/or processes, as these are not central to sign-tracking.

**Figure 1 fig1:**
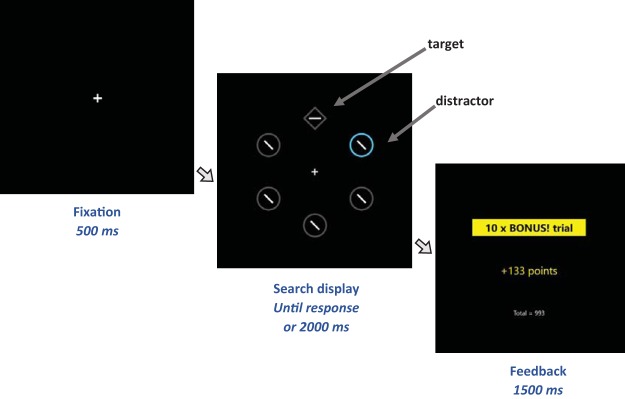
Sequence of trial events in the visual search task. Participants responded to the orientation of the line segment (horizontal or vertical) within the diamond (target). One of the nontarget circles could be a color singleton distractor. Fast, correct responses to the target received monetary reward, depending on the distractor color. A distractor rendered in a high-reward color signaled that this was a bonus trial on which a large reward could be won. If instead the search display contained a distractor rendered in a low-reward color (or did not contain a color singleton distractor), then the trial was a standard trial on which only a small reward was available. Slower response times (RTs) on trials with a high-reward distractor than trials with a low-reward distractor demonstrate value-modulated attentional capture (VMAC).

The task was presented using Inquisit. All stimuli were presented on a black background. Each trial began with a central fixation cross, followed after 500 ms by the search display. The search display comprised six shapes arranged evenly around an imaginary ring (see Figure 1). Five of these shapes were circles, each containing a white line tilted 45° randomly to the left or right. One shape (the *target*) was a diamond containing a line oriented horizontally or vertically. On most trials, one of the circles (termed the *distractor*) was colored; all other shapes were gray. Distractor colors were blue and orange, with assignment of blue and orange to the roles of high-reward and low-reward colors being counterbalanced across participants. Participants’ task was to report the orientation of the line within the target diamond as quickly as possible—by pressing either the C key (horizontal) or M key (vertical)—with faster responses earning more points. The location of the target and distractor, and the orientation of the target’s line segment (vertical or horizontal) were randomly determined on each trial.

Each trial-block of the task comprised 25 trials: 11 trials featuring a distractor rendered in the high-reward color, 11 trials with a distractor in the low-reward color, and three distractor-absent trials (in which all shapes were gray), in random order. For correct responses, on trials with a low-reward distractor and distractor-absent trials, participants won 0.1 points for every ms that their response time (RT) was below 1,000 ms (so a RT of 600 ms would earn 40 points). Trials in which the display contained a high-reward distractor were labeled as bonus trials, and points were multiplied by 10 (so an RT of 600 ms would earn 400 points). Correct responses with RT greater than 1,000 ms and incorrect responses earned no points. The search display remained on-screen until the participant responded or the trial timed-out (after 2,000 ms). A feedback screen then appeared. On “standard” (low-reward distractor or distractor-absent) trials, if the response was correct, feedback showed the number of points earned on that trial; if the response was incorrect, feedback showed “ERROR”; and if the trial timed-out, feedback was “TOO SLOW: Please try to respond faster.” On bonus (high-reward) trials, the corresponding feedback was accompanied by a box labeled “10 × bonus trial!”

Participants were informed that the aim of the visual search task was to earn as many points as possible, and that they could receive a $3 bonus based on their performance. Participants were further informed (a) that when a circle in the high-reward color was present in the search display it would be a bonus trial on which points were multiplied by 10, and (b) that when a circle in the low-reward color was present it would not be a bonus trial. Participants completed five 25-trial blocks, taking a break between blocks; during this break they were shown the total number of points they had earned so far.

Typically, overall accuracy in this type of visual search task is relatively high, and analysis focuses on differences in response time (Anderson, Laurent, & Yantis, 2011a, 2011b; Le Pelley et al., 2015). Therefore, to assess the effect of the reward-signaling distractor on task performance, we calculated a VMAC score for each participant by subtracting mean response time on trials with a low-value distractor from response time on trials with a high-value distractor. A higher VMAC score indicates greater distraction by the high-reward distractor relative to the low-reward distractor; that is, a greater influence of reward on attentional capture. Only correct responses were analyzed. Because we were interested in the effect of reward on steady-state behavior, we calculated VMAC scores using data from the final two blocks (50 trials in total), when participants had had considerable experience of the color–reward relationships—as in previous research using the VMAC task.

### Data Analysis

First, we examined the correlations between each BATCAP behavior scale and its corresponding established scale, to confirm concurrent validity. These between-scale correlations are presented in the supplementary materials. Spearman’s correlations were used as there were a few outliers in the BATCAP scores.

Second, we ran two negative binomial regressions (with robust parameters specified). The two outcome variables were (a) primary addiction-related compulsivity score and (b) primary obsessive–compulsive behavior score. VMAC score was entered as a predictor variable in each regression model. Further, in each regression model, we entered age, gender, Short UPPS-P Impulsivity Scale total score, and DASS-21 score as covariates due to past research showing that these variables can influence compulsive behaviors (Antony, Bieling, Cox, Enns, & Swinson, 1998; Chamberlain, Stochl, Redden, & Grant, 2018; Engel et al., 2016; Verdejo-García, Lawrence, & Clark, 2008), as well as reward-related learning (Anderson, Laurent, & Yantis, 2011b; Anderson, Leal, Hall, Yassa, & Yantis, 2014), and thereby have confounding potential.

Finally, we ran a negative binomial regression, including the above covariates, on OCI–R scores as all participants completed this measure (as opposed to the smaller proportion, *n* = 57, of participants who completed the BATCAP for obsessive–compulsive behaviors). The results for this analysis replicated those found using the BATCAP and are presented in the online supplementary materials.

## Results

Participants were on average 34 years of age (*SD* = 8.5). Forty-three percent were female. Of the sample, 40% (*n* = 57) had engaged in at least one OCD-related behavior in the past month, and 93% (*n* = 133) had engaged in at least one addiction-related behavior. Of the obsessive–compulsive behaviors, checking was predominant (26%), followed by ordering (20%), and contamination-related compulsions (15%). Of the addictive behaviors, excessive Internet use was predominant (83%), followed by alcohol use (51%), gambling (17%), and binge eating (8%).

Across participants, response time in the VMAC task was significantly slower for trials with a high-reward distractor (*M* = 698.6, *SD* = 104.0 ms) than trials with a low-reward distractor (*M* = 682.4, *SD* = 98.5 ms), *t*_142_ = 4.10, *p* < .001, *d*_*z*_ = .34. Accuracy on trials with a high-reward distractor (*M* = 81.7%, *SD* = 11.2%) was significantly lower than on trials with a low-reward distractor (*M* = 83.0%, *SD* = 10.8%), *t*_142_ = 2.00, *p* = .047, *d*_*z*_ = .17. Thus, participants were slower and less accurate on trials with a high-reward distractor, ruling out an interpretation in terms of speed–accuracy trade-off. These findings demonstrate that the high-reward distractor significantly impaired performance on this task relative to the low-reward distractor, demonstrating a VMAC effect. The observed difference in response time (*M* = 16.3 ms) was highly significant with a medium effect size, and is similar in magnitude to that previously reported in laboratory studies using similar procedures (e.g., Anderson et al., 2011a [18 ms]; Le Pelley et al., 2015 [10 ms]), though of more interest for the current study is the ability of this measure to assess individual variation (see below). The current study, which used a reward-only variant of the task in which incorrect responses were not punished, also produced significant evidence for a VMAC effect in response accuracy (though this effect was somewhat weaker).

Each scale of the BATCAP was significantly (or trend level) correlated with the corresponding scale taken from the AUDIT, OCI-R, Young’s Internet Addiction Test, Problem Gambling Severity Index, or the Binge Eating Disorder Screener (with the exception of checking). Correlations are presented in the online supplementary materials. These findings demonstrate the convergent validity of the BATCAP with established measures. These correlations include all participants who completed a measure regardless of whether it was their top-scoring domain or not. Bivariate correlations of outcome variables and covariates with VMAC scores are presented in Table 1. Higher psychological distress was significantly associated with VMAC score (*r*_s_ = .18, *p* = .030), as was impulsivity (*r*_s_ = .17, *p* = .040), supporting the decision to adjust for their influence in the regression analyses.[Table tbl1]

**Figure 2 fig2:**
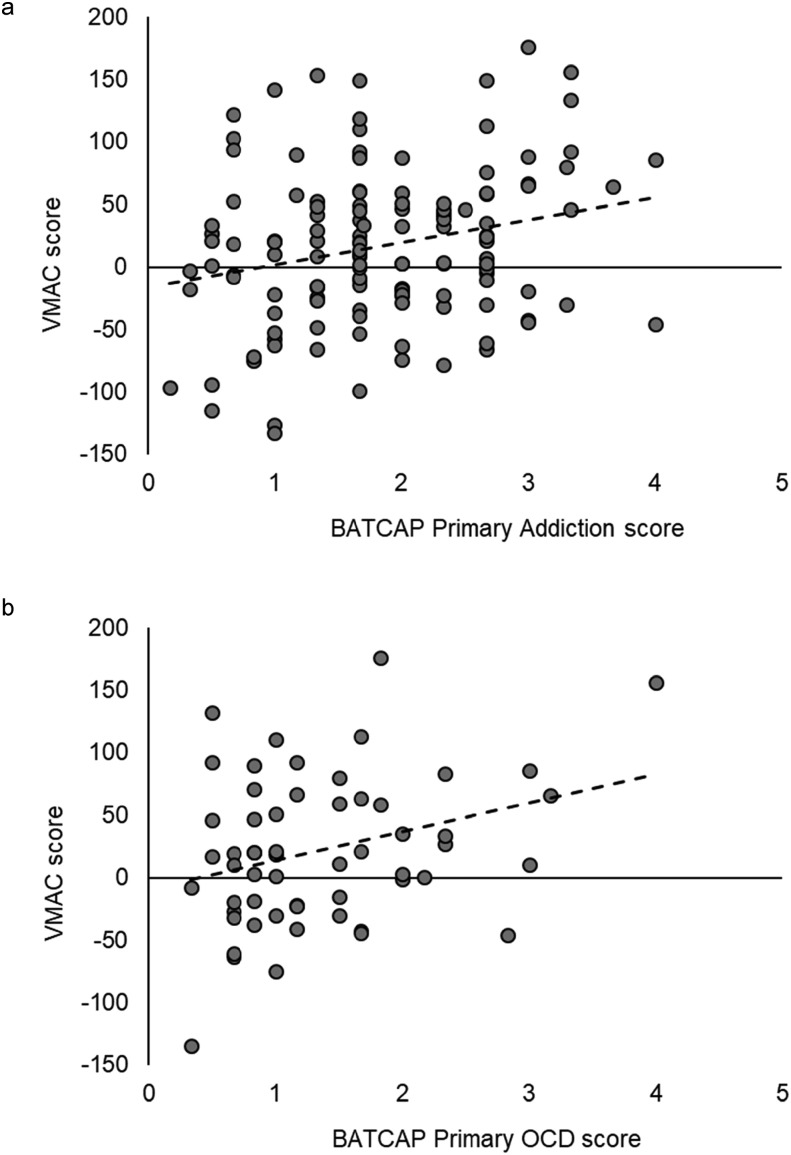
A: A scatterplot of value-modulated attentional capture (VMAC) score (response time [RT] for high minus RT for low) as a function of Brief Assessment Tool for Compulsivity Associated Problems (BATCAP) Primary Addiction score. B: A scatterplot of VMAC score (RT for high minus RT for low) as a function of BATCAP Primary obsessive–compulsive disorder (OCD) score.

Binomial regression results are presented in Table 2. The first binomial regression examined primary addiction-related BATCAP score (the top half of Table 2; *n* = 133). In this model, greater VMAC score was significantly associated with a higher BATCAP score (β = .00, *p* = .043), as was psychological distress (β = .02, *p* < .001). The second binomial regression (the lower half of Table 2; *n* = 57) examined primary OCD-related BATCAP score. In this model, greater VMAC score was significantly associated with a higher primary BATCAP score (β = .00, *p* = .020) as was female gender (β = .35, *p* = .014). Figure 2A and 2B show a scatterplot of VMAC score as a function of BATCAP addiction-related and OCD-related primary score, respectively.[Table tbl2][Fig fig2]

## Discussion

The current study found that performance in the value-modulated attentional capture (VMAC) task was sensitive to compulsivity, transdiagnostically. That is, across addictive and OCD-related behaviors, participants who showed greater compulsivity scores also showed more attention toward signals of high reward even though such attention was at the expense of procuring the reward. Phrased differently, more compulsive participants exhibited evidence of greater attentional control by reward-signaling stimuli. The finding that VMAC was significantly associated with severity of compulsivity across primary addictive and OCD-related behaviors viewed separately from each other is consistent with the idea that VMAC measures compulsivity risk, possibly mediated by enhanced incentive salience attribution toward Pavlovian cues promoting maladaptive cue-triggered behaviors. Although research has linked individual differences in incentive salience attribution to addiction risk (Albertella et al., 2017; Flagel et al., 2009), this is the first study to do so in relation to compulsive behaviors transdiagnostically, including severity of obsessive–compulsive behaviors. These findings highlight the potential of value-modulated attentional capture as a measure that is sensitive to different clinical manifestations of compulsivity, including behaviors that might not be so obvious in their link to reward-related learning processes.

As explained in the introduction, a predisposition toward incentive salience attribution could contribute to maladaptive, cue-triggered behaviors, such as those seen in addictions and OCD, by rendering Pavlovian signals for reward or safety attractive in their own right, able to elicit self-directed approach responses. In addictions, incentive salience may be attributed not only to cues associated with the rewarding effects of the drug but also to those associated with relief from withdrawal and/craving, whereas in OCD incentive salience is likely to accrue primarily to cues associated with relief from anxiety. An implication of incentive salience accruing to reward and safety signals is that drug-taking and compulsive behaviors may be resistant to treatment precisely because such signals have become attractive in their own right. Finally, the incentive salience account of such behaviors shares similarities with the idea that drug seeking/taking and OCD reflect a predisposition toward habit formation and are characterized by abnormalities in the underlying stimulus–response associations (Belin, Belin-Rauscent, Murray, & Everitt, 2013; Everitt & Robbins, 2005; Gillan, Robbins, Sahakian, van den Heuvel, & van Wingen, 2016; Voon et al., 2015). However, the current model differs by proposing that abnormalities exist in how knowledge of stimulus–reward relationships influences fundamental processes of attention (i.e., incentive salience attribution), which in turn promotes maladaptive stimulus-driven behavior.

An alternative explanation for the finding of greater VMAC being associated with greater problematic behaviors is that it is the behaviors themselves that have led to enhanced VMAC. For instance, with regard to addictive behaviors, the relationship between VMAC and severity of compulsive behaviors might be the result of repeated exposure to addiction-related behaviors and/or substance use having sensitized incentive salience processes (as proposed by the incentive salience model of addiction; Robinson & Berridge, 2000). This sensitization has been shown to transfer to general (nondrug) reward cues. For instance, one study found that rats sensitized through amphetamine exposure subsequently showed enhanced sign-tracking toward stimuli that predicted sucrose (Wyvell & Berridge, 2001). In another study, exposure to cannabinoids in early adolescence resulted in greater sign-tracking toward food-related cues in adulthood, long after cannabinoid exposure had ceased (Schoch et al., 2018). The association between VMAC and OCD-related compulsivity might be explained in the same way, but through the involvement of stress-induced sensitization processes, which are closely related to drug-induced sensitization processes (Kalivas & Stewart, 1991). Indeed, sign-tracking in animals has been shown to predict the development of sensitization following drug treatment (Flagel, Watson, Akil, & Robinson, 2008). Thus, enhanced VMAC in the context of subclinical addiction-related or obsessive–compulsive behaviors might index a vulnerability toward progressing to more persistent expressions of compulsivity following exposure to high levels of stress and/or substance use.

Finally, the current findings support the BATCAP as a potentially useful screening tool for the assessment of compulsive behaviors; a tool that can easily be modified to suit most behaviors of interest. Importantly, it allows the measurement of aspects of compulsivity across different types of behaviors so that they may be selected according to most problematic (as done here) or over time to see if compulsivity in one domain progresses to others. Examining aspects of compulsivity, like primary domains or cumulative severity across different behaviors (which can reveal patterns of progression, sensitization, and comorbidity), can only be achieved using scales that allow such comparisons. Regarding cumulative severity, the brevity of the BATCAP means that it could be administered across all participants (and not just current endorsers), across different behaviors, allowing future investigations of this nature.

The present study has several limitations. First, it was conducted online, with the limitation that context and conditions could not be tightly controlled. However, web-based methods of delivering cognitive tests have shown comparable results to laboratory-based ones (McGraw, Tew, & Williams, 2000). That said, the drop-out rate was relatively high. This may reflect Internet connectivity issues, greater likelihood of environmental distractions during testing (vs. in-person assessment in a quiet controlled room), and the length of assessment (which was not unusual for laboratory-based research of this type but was relatively long for a web-based study). Another limitation, which may have contributed to attrition, is that the questionnaires and VMAC task were administered via different websites, with participants required to follow a link to move from one to the other. Future work could minimize attrition by using a more focused (i.e., shorter) battery and deploying questionnaires and cognitive testing on one platform. The online procedure—with unsupervised participants, some of whom may have been less attentive and motivated than in a lab setting (see Oppenheimer, Meyvis, & Davidenko, 2009)—may also have contributed to the relatively high number of participants (around 15%) who did not perform above chance (50%) accuracy in the VMAC task. As a conservative measure, we excluded data from these participants. Mean accuracy for remaining participants was relatively high (>80% correct), and we note that our web-based procedure was clearly sensitive to the effect of reward on attention, replicating the VMAC effect in response times observed in previous lab-based studies (e.g., Le Pelley et al., 2015). Hence this task is viable as an online procedure. In fact, a relatively high error rate in the current study is understandable given that we used a variant of the task in which incorrect responses were not punished, and this may explain why we also observed a significant VMAC effect in accuracy. For consistency with previous work, our current analyses of VMAC focused on response time, but future studies using this task may benefit from exploring how errors themselves are related to compulsivity and whether VMAC expressed in response time and/or errors represents different profiles. With regard to the BATCAP, although this measure has yet to be validated systematically, it had excellent concurrent validity against established measures, especially for the addictions. Nonetheless, future studies validating the BATCAP will help clarify the processes involved in driving the current findings. Given the brief nature of the BATCAP, it could be modified in future to capture information about current and past symptoms/behaviors (rather than only current). Finally, this study was cross-sectional and as such is limited by the issues that apply to cross-sectional research.

In conclusion, the current study found that reward-related attentional capture may reflect transdiagnostic risk-related traits, such as a predisposition toward the display of repetitive and maladaptive cue-triggered behavior. These findings have implications for understanding the mechanisms that underlie compulsive behaviors and highlight reward-related attentional capture as a promising transdiagnostic cognitive risk marker for compulsivity.

## Supplementary Material

10.1037/adb0000484.supp

## Figures and Tables

**Table 1 tbl1:** Bivariate Correlations of VMAC With DASS-21, S-UPPS-P, and BATCAP Scores

Statistic	VMAC	BATCAP Addiction	BATCAP OCD	DASS-21	S-UPPS-P
*r*_*s*_	—	.21	.23	.18	.17
*p*	—	.017	.090	.030	.040
*N*	—	133	57	143	143
*M*/median	14.8	16.7	11.7	27	9.3
*SD*/range	61.29	2–40	3–30	21–78	5–15
*Note*. VMAC = value-modulated attentional capture; BATCAP = Brief Assessment Tool for Compulsivity Associated Problems; OCD = obsessive–compulsive disorder; DASS-21 = brief Depression Anxiety Stress Scales; S-UPPS-P = Short UPPS-P Impulsivity Scale.					

**Table 2 tbl2:** Regression Results of Negative Binomial Regressions on BATCAP Scores

Dependent variable	B	*SE*	Wald χ^2^	*p*
Primary addiction-related BATCAP score (*n* = 133)				
Age	.001	.0042	.04	.844
Gender	−.112	.0817	1.88	.170
VMAC	.001	.0007	4.10	.043
DASS-21	.014	.0025	30.98	<.001
S-UPPS-P	−.003	.0188	.03	.874
Primary OCD-related BATCAP score (*n* = 57)				
Age	.005	.0082	.43	.510
Gender	.345	.1409	5.99	.014
VMAC	.003	.0013	5.44	.020
DASS-21	.009	.0057	2.34	.126
S-UPPS-P	.027	.0483	.31	.578
*Note*. VMAC = value-modulated attentional capture; BATCAP = Brief Assessment Tool for Compulsivity Associated Problems; OCD = obsessive–compulsive disorder; DASS-21 = brief Depression Anxiety Stress Scales; S-UPPS-P = Short UPPS-P Impulsivity Scale.				

## Footnotes

1 The study reimbursement was advertised as 6 USD with the potential of an additional 3 USD bonus based on VMAC performance. All participants who completed the study actually received 9 USD.

2 The BATCAP internet use scale comprised three items (items included were time spent, anxiety if prevented, and strongest urge). Therefore, we used the averages of each subscale to make the scores comparable.
