# Supplementary material for: Reward-Related Attentional Capture Is Associated With Severity of Addictive and Obsessive–Compulsive Behaviors
Source: Psychol Addict Behav. 2019 Jun 20;33(5):495–502. doi: 10.1037/adb0000484 (PMC6681689; doi:10.1037/adb0000484)
Supplement: Supplementary file 1 [file adb0000484Supplemental.docx]

**SUPPLEMENTARY MATERIALS**

**TO ACCOMPANY**

**Reward-related attentional capture is associated with severity of addictive and obsessive-compulsive behaviors**

This document has 3 sections:

1. BATCAP items
2. Table S1. Correlation analyses
3. Table S2. Regression results for OCI-R scores

# 1. BATCAP items

**Part 1** (used for filtering)

Do you or have you ever [describe behavior/s]?

- Never

- Yes, but not in the past month

- Yes, including in the past month *

* Thinking about the last month, how often did you perform these behaviors?

- 1-4 days in the past month

- 2-3 days per week

- 4-5 days per week

- 6-7 days per week

**Part 2** (used for scoring)

For the questions that follow, the behaviours described above are referred to as 'these behaviours'.

In the past week**:**

|  | 0 | 1 | 2 | 3 | 4 |
| --- | --- | --- | --- | --- | --- |
| a) On average, how much time was occupied by these behaviors? | None | Less than 1hr/day | 1-3 hrs/day | 3-8 hrs/day | Over 8 hrs a day |
| b) How much distress did these behaviors cause you? | None | Mild distress | Moderate distress | Severe distress | Extreme distress (disabling) |
| c) How hard was it for you to control these behaviors? | Not at all / I had complete control | Somewhat / I had much control | Moderately hard / I had moderate control | Very hard / I had little control | Extremely hard / I had no control |
| d) How much did these behaviors interfere with work/school, social, or family life? | Not at all | Slight interference | Moderate interference | Much interference | Extreme interference (disabling) |
| e) How anxious would you become if prevented from these behaviors? | No anxiety | Only slightly anxious | Anxiety would mount but remain manageable | Prominent and very disturbing increase in anxiety | Incapacitating anxiety |
| f) At its most severe point (in the past week), what was the strength of your strongest urge/craving to perform these behaviors? | I did not experience any urge/ craving | Mild | Moderate | Strong | Extremely strong |

# 2. Table S1. Correlation analyses

|  | | | | | | | | | | | | | | | | |
| --- | --- | --- | --- | --- | --- | --- | --- | --- | --- | --- | --- | --- | --- | --- | --- | --- |
|  |  | 1 | 2 | 3 | 4 | 5 | 6 | 7 | 8 | 9 | 10 | 11 | 12 | 13 | 14 | 15 |
| 1. VMAC score | r_s_ | 1.00 |  |  |  |  |  |  |  |  |  |  |  |  |  |  |
|  | p |  |  |  |  |  |  |  |  |  |  |  |  |  |  |  |
|  | n | 143 |  |  |  |  |  |  |  |  |  |  |  |  |  |  |
| 2. OCI-R Checking | r_s_ | .07 | 1.00 |  |  |  |  |  |  |  |  |  |  |  |  |  |
|  | p | .377 |  |  |  |  |  |  |  |  |  |  |  |  |  |  |
|  | n | 143 | 143 |  |  |  |  |  |  |  |  |  |  |  |  |  |
| 3. OCI-R Ordering | r_s_ | .12 | .60 | 1.00 |  |  |  |  |  |  |  |  |  |  |  |  |
|  | p | .141 | .000 |  |  |  |  |  |  |  |  |  |  |  |  |  |
|  | n | 143 | 143 | 143 |  |  |  |  |  |  |  |  |  |  |  |  |
| 4. OCI-R Washing | r_s_ | .17 | .59 | .52 | 1.00 |  |  |  |  |  |  |  |  |  |  |  |
|  | p | .037 | .000 | .000 |  |  |  |  |  |  |  |  |  |  |  |  |
|  | n | 143 | 143 | 143 | 143 |  |  |  |  |  |  |  |  |  |  |  |
| 5. IAT | r_s_ | .15 | .39 | .36 | .34 | 1.00 |  |  |  |  |  |  |  |  |  |  |
|  | p | .094 | .000 | .000 | .000 |  |  |  |  |  |  |  |  |  |  |  |
|  | n | 118 | 118 | 118 | 118 | 118 |  |  |  |  |  |  |  |  |  |  |
| 6. AUDIT | r_s_ | .15 | -.02 | .05 | -.11 | .11 | 1.00 |  |  |  |  |  |  |  |  |  |
|  | p | .104 | .800 | .625 | .235 | .277 |  |  |  |  |  |  |  |  |  |  |
|  | n | 123 | 123 | 123 | 123 | 118 | 123 |  |  |  |  |  |  |  |  |  |
| 7. PGSI | r_s_ | .13 | .11 | .07 | .36 | .45 | .04 | 1.00 |  |  |  |  |  |  |  |  |
|  | p | .560 | .622 | .729 | .085 | .049 | .864 |  |  |  |  |  |  |  |  |  |
|  | n | 24 | 24 | 24 | 24 | 20 | 23 | 24 |  |  |  |  |  |  |  |  |
| 8. BEDS | r_s_ | .29 | .23 | .37 | .18 | -.28 | .05 |  | 1.00 |  |  |  |  |  |  |  |
|  | p | .369 | .468 | .239 | .565 | .506 | .891 |  |  |  |  |  |  |  |  |  |
|  | n | 12 | 12 | 12 | 12 | 8 | 10 | < 5 | 12 |  |  |  |  |  |  |  |
| 9. BC - Order | r_s_ | .15 | .05 | **.33** | .37 | .78 | .24 | .54 | .32 | 1.00 |  |  |  |  |  |  |
|  | p | .449 | .794 | **.090** | .049 | .000 | .274 | .456 | .684 |  |  |  |  |  |  |  |
|  | n | 28 | 28 | **28** | 28 | 18 | 23 | < 5 | < 5 | 28 |  |  |  |  |  |  |
| 10. BC - Check | r_s_ | .24 | **.25** | .38 | .48 | .54 | -016 |  |  | .38 | 1.00 |  |  |  |  |  |
|  | p | .147 | **.131** | .019 | .003 | .006 | .417 |  |  | .221 |  |  |  |  |  |  |
|  | n | 37 | **37** | 37 | 37 | 24 | 29 | < 5 | < 5 | 12 | 37 |  |  |  |  |  |
| 11. BC - Wash | r_s_ | .34 | .28 | .05 | **.54** | .19 | .18 |  |  | .63 | .28 | 1.00 |  |  |  |  |
|  | p | .125 | .206 | .833 | **.009** | .541 | .477 |  |  | .070 | .358 |  |  |  |  |  |
|  | n | 22 | 22 | 22 | **22** | 13 | 17 | < 5 | < 5 | 9 | 13 | 22 |  |  |  |  |
| 12. BC - Alc | r_s_ | .16 | .09 | -.02 | .11 | -.04 | **.53** | .11 |  | -.18 | -.09 | .09 | 1.00 |  |  |  |
|  | p | .178 | .473 | .886 | .359 | .765 | **.000** | .705 |  | .530 | .701 | .785 |  |  |  |  |
|  | n | 73 | 73 | 73 | 73 | 65 | **73** | 15 | < 5 | 14 | 19 | 11 | 73 |  |  |  |
| 13. BC - Gamble | r_s_ | .22 | .24 | .02 | .09 | .37 | .25 | **.64** |  | -.78 |  |  | .32 | 1.00 |  |  |
|  | p | .295 | .250 | .921 | .684 | .104 | .243 | **.001** |  | .222 |  |  | .239 |  |  |  |
|  | n | 24 | 24 | 24 | 24 | 20 | 23 | **24** | < 5 | < 5 | < 5 | < 5 | 15 | 24 |  |  |
| 14. BC - Internet | r_s_ | .17 | .35 | .37 | .34 | **.60** | -.02 | .25 | .21 | .41 | .53 | .16 | .01 | .27 | 1.00 |  |
|  | p | .071 | .000 | .000 | .000 | **.000** | .826 | .287 | .616 | .087 | .008 | .601 | .913 | .241 |  |  |
|  | n | 118 | 118 | 118 | 118 | **118** | 104 | 20 | 8 | 18 | 24 | 13 | 65 | 20 | 118 |  |
| 15. BC - Binge Eat. | r_s_ | .37 | .33 | .34 | .26 | -.08 | .33 |  | **.88** |  |  |  |  |  | .14 | 1.00 |
|  | p | .233 | .298 | .278 | .423 | .843 | .358 |  | **.000** |  |  |  |  |  | .736 |  |
|  | n | 12 | 12 | 12 | 12 | 8 | 10 | < 5 | **12** | < 5 | < 5 | < 5 | < 5 | < 5 | 8 | 12 |
| ‘BC’: BATCAP | | | | | | | | | | | | | | | | |

3. **Table S2**. Regression results. DV: OCI-R scores (*N* = 143)

|  | B | SE | Wald Χ^2^ | p |
| --- | --- | --- | --- | --- |
| Age | -.013 | .0085 | 2.205 | .138 |
| Gender | .125 | .1474 | .719 | .397 |
| VMAC | **.002** | **.0011** | **5.172** | **.023** |
| DASS-21 | **.034** | **.0053** | **41.180** | **.000** |
| S-UPPS-P | .007 | .0345 | .041 | .840 |
